# The role of artificial root exudate components in facilitating the degradation of pyrene in soil

**DOI:** 10.1038/s41598-017-07413-3

**Published:** 2017-08-02

**Authors:** Hainan Lu, Jianteng Sun, Lizhong Zhu

**Affiliations:** 1 Department of Environmental Science, Zhejiang University, Hangzhou Zhejiang, 310058 China; 2 Zhejiang Provincial Key Laboratory of Organic Pollution Process and Control, Hangzhou Zhejiang, 310058 China

## Abstract

Root exudates play an important role in the phytoremediation of soils contaminated by organic pollutants, but how root exudate components affect the remediation process is not well understood. In this study, we explored the effects and mechanisms of the major root exudates, including glucose, organic acids, and serine, in the rhizoremediation of pyrene-contaminated soil. The results showed that glucose increased the degradation of pyrene (54.3 ± 1.7%) most significantly compared to the organic acids (45.5 ± 2.5%) and serine (43.2 ± 0.1%). Glucose could significantly facilitate the removal of pyrene in soil through promoting dehydrogenase activity indicated by a positive correlation between the removal efficiency of pyrene and the soil dehydrogenase activity (*p* < 0.01). Furthermore, root exudates were able to change soil microbial community, particularly the bacterial taxonomic composition, thereby affecting the biodegradation of pyrene. Glucose could alter soil microbial community and enhance the amount of *Mycobacterium* markedly, which is dominant in the degradation of pyrene. These findings provide insights into the mechanisms by which root exudates enhance the degradation of organic contaminants and advance our understanding of the micro-processes involved in rhizoremediation.

## Introduction

Phytoremediation is a green and cost-effective remediation approach in which plants are used to purify contaminated soils and sediments^1^. Many plants (alfalfa, tall fescue, ryegrass, etc.) have been used to remediate soil contaminated with organic pollutants such as polycyclic aromatic hydrocarbons (PAHs), petroleum, and polychlorinated biphenyls (PCBs)^2–7^. However, only a small percentage of the organic pollutants are degraded by plants, while most soil organic pollutants are degraded by the microorganisms in the rhizosphere^8, 9^. Thus, rhizoremediation is an important aspect of phytoremediation^10–14^.

Rhizoremediation has great potential for mitigating organic pollution in soils^15, 16^. Reilley found that the degradation of PAHs increased with an increased density of microbes in the rhizosphere^17^. The density of bacteria in the rhizosphere increased by 2–4 fold^18, 19^, indicating that the bacteria in the rhizosphere exhibited stronger metabolic capabilities than the bacteria in the non-rhizosphere soil. Additionally, some studies found that ryegrass root exudates induced shifts in the spatial pattern of microbial communities and noted that the degradation of organic pollutants in soil mainly occurs in the rhizosphere^20, 21^. The mechanisms by which root exudates enhance degradation of organic pollutions involve the activation of microbial enzymatic pathways^22^, enhanced bioavailability of organic pollutions^23–25^, and increased abundance of degradation bacteria^15, 26^. Nevertheless, the degradation of organic pollutants in the soil rhizosphere is attributed to the joint actions of root exudates and microorganisms^15, 27, 28^. However, how the components of root exudates affect microbial activity and, in turn, the biodegradation of organic pollutants remains unclear.

Root exudates are primarily photosynthetic products that are transferred from the roots and released into the rhizosphere^29, 30^. Plant roots can release a wide range of compounds as root exudates^31^, including carbohydrates, organic acids, amino acids, mucilages, phenolic compounds, fatty acids, sterols, and vitamins^32^. Some studies considered that root exudates can serve rhizosphere microorganisms as nutrient sources, in which low-molecular-weight (LMW) substances, such as carbohydrates, organic acids, and amino acids, played a key role in pollutant degradation^28, 33–35^. However, few studies have evaluated the specific role of individual root exudate components in rhizoremediation. Although an association between root exudates and the enhanced degradation of organic pollutants has been observed^33^, the predominant functional components have not been identified, and the degradation mechanism remains unclear.

The aim of this study was to explore the underlying mechanisms of the key root exudate components mainly responsible for the accelerated rhizodegradation of pyrene, thereby revealing the responses of soil microorganisms to different root exudate components. The findings of this study are expected to advance our understanding of the interactions between root exudates and soil microorganisms during rhizoremediation.

## Results

### Dissipation of pyrene in polluted soil

**Figure 1:**
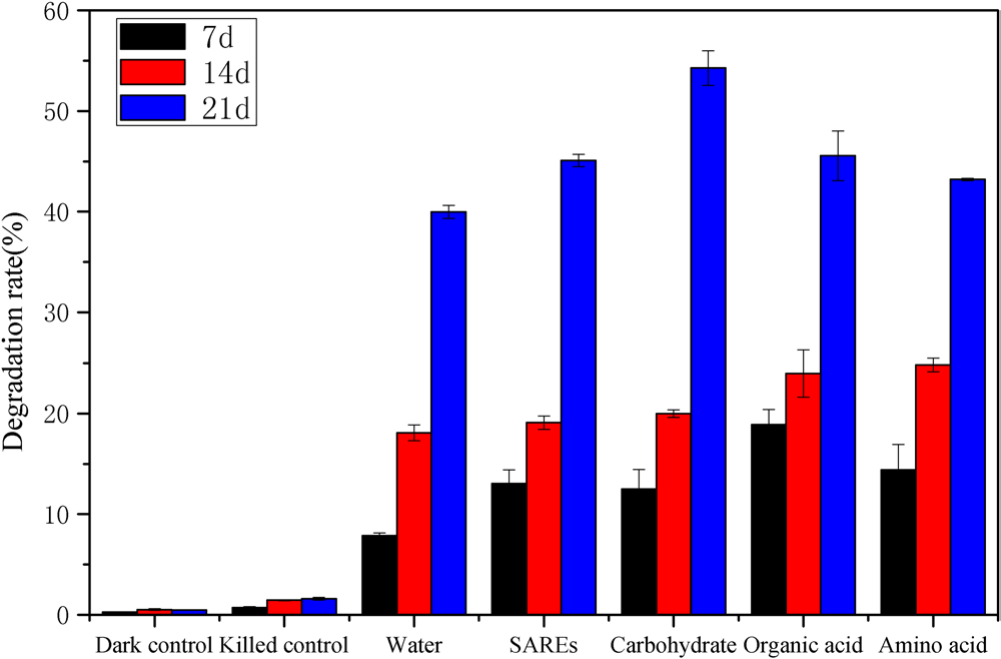
Effect of root exudate components on the degradation rate of pyrene.

The degradation rates of pyrene in polluted soils are depicted in Fig. 1. The addition of root exudate components increased the degradation rate of pyrene in different periods. After 7 days of cultivation, a significantly increased degradation rate of pyrene was observed in the organic acid treatment (18.9 ± 1.5%) compared with that in the control (7.9 ± 0.3%). After 21 days of cultivation, pyrene degradation was all promoted by the addition of simplified artificial root exudates (SAREs) or different root exudate components compared with that in the control (39.9 ± 0.7%). The percentages of biodegraded pyrene in the SAREs, carbohydrate, organic acid, and amino acid treatments were 45.1 ± 0.6%, 54.3 ± 1.7%, 45.5 ± 2.5%, and 43.2 ± 0.1%, respectively. The level of pyrene degradation in the carbohydrate treatment was significantly higher than that in the control by approximately 36.1%. The carbohydrate treatment had the highest level of pyrene degradation, followed by the organic acid treatment.

### Effect of root exudate components on soil enzymatic activities

**Figure 2:**
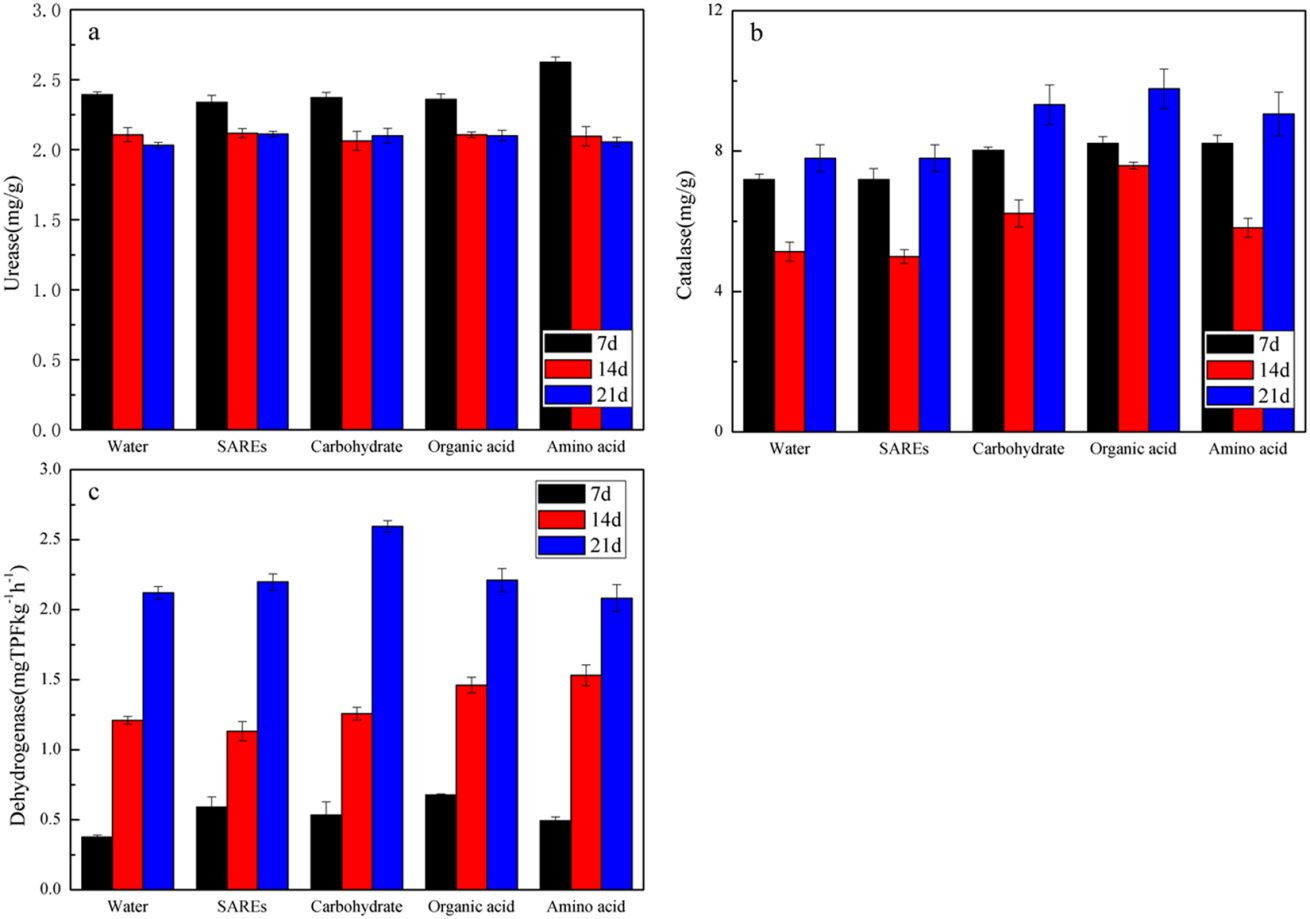
Effect of root exudate components on urease (**a**), catalase (**b**), and dehydrogenase (**c**) activities in different cultivation times.

Three types of enzymes, i.e., urease, catalase and dehydrogenase, were selected to estimate the changes in nitrogen utilization and the microbial responses in soil during the experiment. At cultivation times of 7, 14 and 21 days, the activities of these soil enzymes were determined. As shown in Fig. 2, their activities were different. No significant changes in urease activity were observed among the treatments or among different periods, except that the urease activity in the amino acid treatment achieved the maximal value (2.62 ± 0.04 mg/g) at 7 days (Fig. 2a). The changes in catalase activity were different from the changes in urease activity (Fig. 2b). In every period, the root exudate components increased the catalase activity. The catalase activity in all treatments was reduced at 14 days but increased at 21 days. At 21 days, the catalase activity was 7.79 ± 0.38 mg/g in the control. The organic acid treatment resulted in the largest increase in catalase activity (to 9.77 ± 0.57 mg/g), whereas the catalase activity increased to 9.32 ± 0.56 mg/g and 9.05 ± 0.62 mg/g with the addition of the carbohydrate and amino acid, respectively. The change in dehydrogenase activity was different from the change in catalase activity. In each treatment, the dehydrogenase activity increased as the culture time increased (Fig. 2c). During the first 7 days, the addition of the carbohydrate, organic acids and amino acid increased the dehydrogenase activity to 0.53 ± 0.09, 0.67 ± 0.01, and 0.49 ± 0.03 mgTPFkg^−1^ h^−1^ respectively, compared to the control (0.37 ± 0.02 mgTPFkg^−1^ h^−1^). After 21 days, the addition of the carbohydrate led to the largest increase in dehydrogenase activity (reaching 2.59 ± 0.04 mgTPFkg^−1^ h^−1^) compared to the control (reaching 2.12 ± 0.04 mgTPFkg^−1^ h^−1^).

### Differences in microbial phospholipid fatty acids (PLFAs)

**Figure 3:**
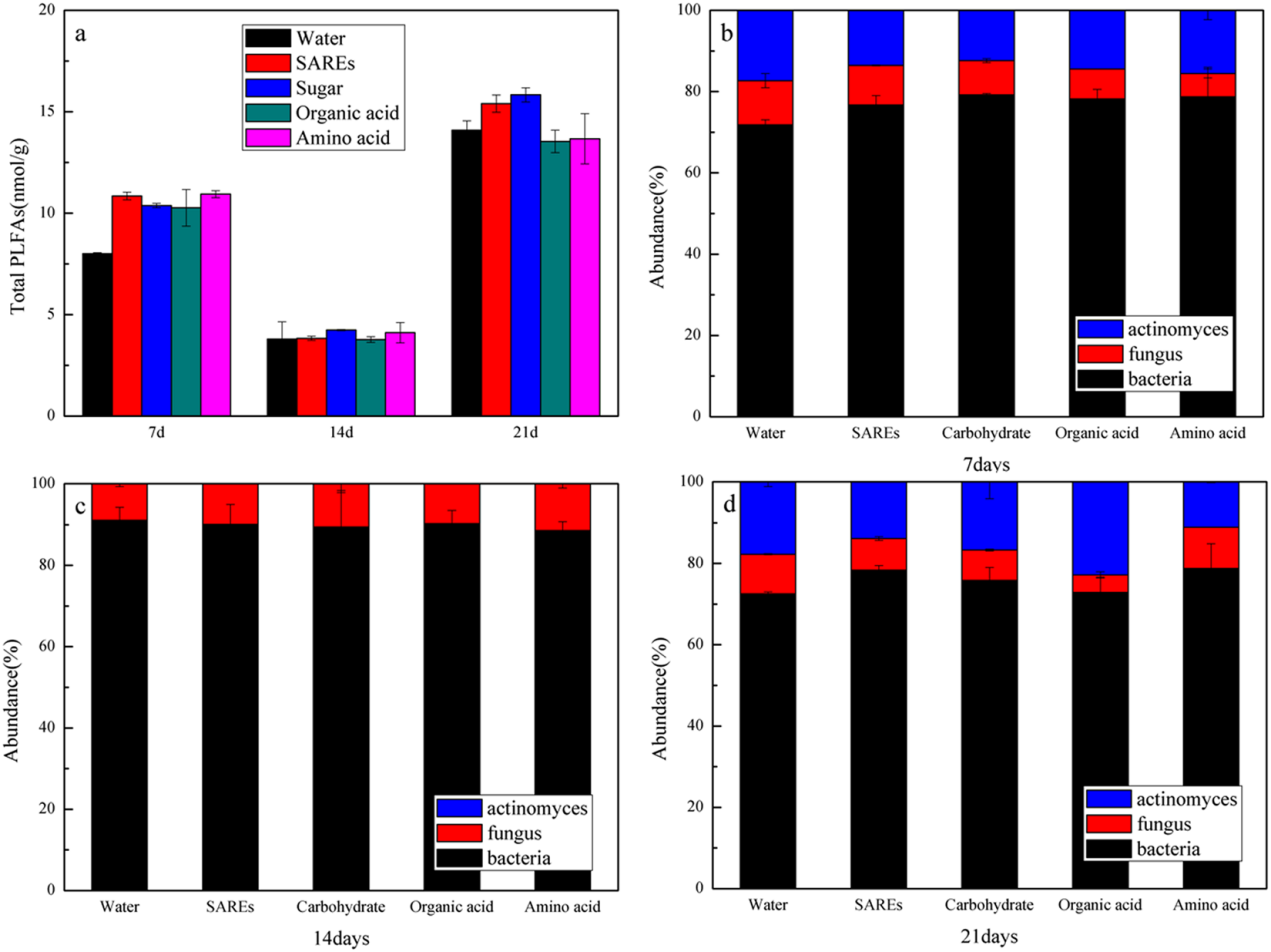
Effect of root exudate components on microbial biomass changes (**a**) and the microbial communities of bacteria, fungi and actinomycetes in different cultivation times, 7 days (**b**), 14 days (**c**) and 21 days (**d**).

PLFA analysis is a commonly used method for characterizing microbial communities^36–39^. PLFAs were chosen as a relative indicator of microbial biomass throughout the experiment, and the microbial biomass in each treatment did not increase with increasing incubation time. At 7 days, the microbial biomass was 10.26 ± 0.90–10.94 ± 0.17 nmol/g when the components of root exudates were added and 8.01 ± 0.04 nmol/g in the control. Then, the microbial biomass decreased to 3.77 ± 0.14–4.24 ± 0.03 nmol/g in each treatment. Although the microbial biomass did not significantly differ among the treatments, it was significantly increased at 21 days, reaching 14.09 ± 0.46 nmol/g in the control and 13.32 ± 0.55–15.83 ± 0.35 nmol/g in the root exudate treatments (Fig. 3a). Among the treatments, significant changes were observed in the carbohydrate and organic acid treatments. The PLFAs of bacteria, fungi and actinomycetes accounted for 71.8 ± 1.2–78.7 ± 7.3%, 5.8 ± 1.1–10.8 ± 1.7% and 12.4 ± 0.5–17.3 ± 0.1% of the total PLFAs of soil microbes at 7 days, respectively (Fig. 3b). However, at 14 days, the PLFAs of bacteria and fungi accounted for 88.5 ± 2.2–91.1 ± 3.2% and 8.8 ± 0.7–11.5 ± 1.0% of the total PLFAs, respectively, and actinomycetes were not detected (Fig. 3c). At the end of the study (21 days), the PLFAs of bacteria, fungi and actinomycetes accounted for 71.5 ± 0.5–78.8 ± 6.0%, 4.3 ± 0.7–10.1 ± 0.1% and 11.0 ± 0.2–22.8 ± 0.2%, respectively, of the total PLFAs (Fig. 3d). Throughout the experiment, bacteria obviously dominated the soil microbial community.

### Bacterial diversity analysis

The 16S rRNA sequencing generated a range of 53204 ± 16734 to 101526 ± 33776 sequences per sample (Table 1), and a range of 2150 ± 2 to 2417 ± 18 operational taxonomic units (OTUs) numbers was identified. Meanwhile, rarefaction curve analysis supported the generally high full extent of microbial diversity detected in this study. α-Diversity analysis revealed higher values in all treatments (Table 1). The Chao1 and Shannon indices, as well as higher Simpson indexes, were determined, indicating higher bacterial richness and diversity at 14 days. Moreover, the values determined for the organic acid and carbohydrate treatments were lower than those for the control in the different periods.

**Table 1:** Richness, diversity and sample coverage for high-throughput sequencing with bacterial 16S rRNA gene libraries of soil microorganisms.

Sample Name	Sample Size	OTUs Number	shannon	simpson	chao1	Coverage
7d.C	94081 ± 19234	2359 ± 111	8.94 ± 0.02	0.993 ± 0.001	2432 ± 35	0.99
7d.O	90528 ± 12494	2330 ± 16	8.87 ± 0.15	0.992 ± 0.002	2409 ± 92	0.99
7d.S	68719 ± 14390	2164 ± 114	8.90 ± 0.02	0.993 ± 0.001	2373 ± 28	0.99
14d.C	53204 ± 16734	2170 ± 183	9.22 ± 0.09	0.995 ± 0.001	2497 ± 64	0.99
14d.O	67239 ± 11824	2271 ± 122	9.06 ± 0.11	0.994 ± 0.001	2531 ± 184	0.99
14d.S	56956 ± 16967	2187 ± 146	9.10 ± 0.02	0.994 ± 0.001	2422 ± 25	0.99
21d.C	57036 ± 7048	2150 ± 2	8.94 ± 0.12	0.992 ± 0.001	2475 ± 97	0.99
21d.O	101526 ± 33776	2417 ± 18	8.88 ± 0.24	0.991 ± 0.003	2482 ± 131	0.99
21d.S	77867 ± 68283	2212 ± 616	9.02 ± 0.23	0.993 ± 0.002	2399 ± 267	0.97

7d: 7 days, 14d: 14 days, 21d: 21 days, C: control, O: organic acid, S: carbohydrate.

The bacterial taxonomic compositions in the control and treatments were significantly different (Table S1). The most frequently detected bacteria in the controls were *Proteobacteria* (23.7 ± 3.0–28.4 ± 4.0%), *Acidobacteria* (16.5 ± 5.3–24.1 ± 2.0%), *Actinobacteria* (14.1 ± 3.1–18.1 ± 2.5%), *Gemmatimonadetes* (10.0 ± 0.3–13.2 ± 0.0%), and *Chloroflexi* (9.2 ± 2.3–15.7 ± 0.2%). Meanwhile, the other treatments revealed similar distribution patterns of the major bacterial phyla, with *Proteobacteria*, *Acidobacteria*, *Actinobacteria*, *Gemmatimonadetes*, and *Chloroflexi* constituting 24.1 ± 0.7–31.5 ± 2.1%, 16.4 ± 1.2–22.9 ± 0.2%, 12.0 ± 1.3–21.2 ± 0.1%, 9.8 ± 0.2–11.9 ± 0.5%, and 7.5 ± 0.9–10.9 ± 0.1% of the total bacterial population, respectively.

**Figure 4:**
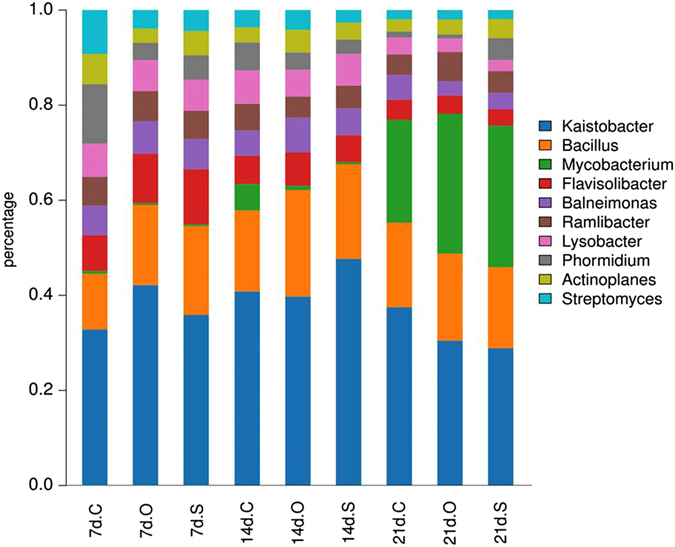
Biodiversity of 10 dominant bacterial genera expressed as relative abundance (%) of OTUs in soil.

At the genus level, more than 170 genera were detected using 16S rRNA sequencing. In the control, 16S rRNA sequencing revealed 141 genera, and 127 genera were detected in the reference soils. At 7 days, members from *Kaistobacter* (34.2 ± 2.3–38.8 ± 4.6%), *Bacillus* (11.6 ± 0.1–19.6 ± 3.7%), *Flavisolibacter* (8.7 ± 1.8–10.6 ± 1.4%), *Balneimonas* (4.7 ± 2.2–5.5 ± 1.8%), *Lysobacter* (6.5 ± 0.7–7.3 ± 1.1%), and *Phormidium* (3.0 ± 0.9–12.6 ± 2.3%) were dominant across different communities, and similar relative abundance distributions of the dominant bacterial genera were observed at 14 days. However, at 21 days, the bacterial community changed and mainly consisted of *Kaistobacter* (30.6 ± 2.5–41.2 ± 5.3%), *Mycobacterium* (22.2 ± 0.8–28.9 ± 0.7%), *Bacillus* (14.8 ± 4.3–22.5 ± 7.7%), *Balneimonas* (2.4 ± 1.5–3.5 ± 2.4%), and *Ramlibacter* (4.2 ± 0.5–5.2 ± 1.3%) (Fig. 4).

**Figure 5:**
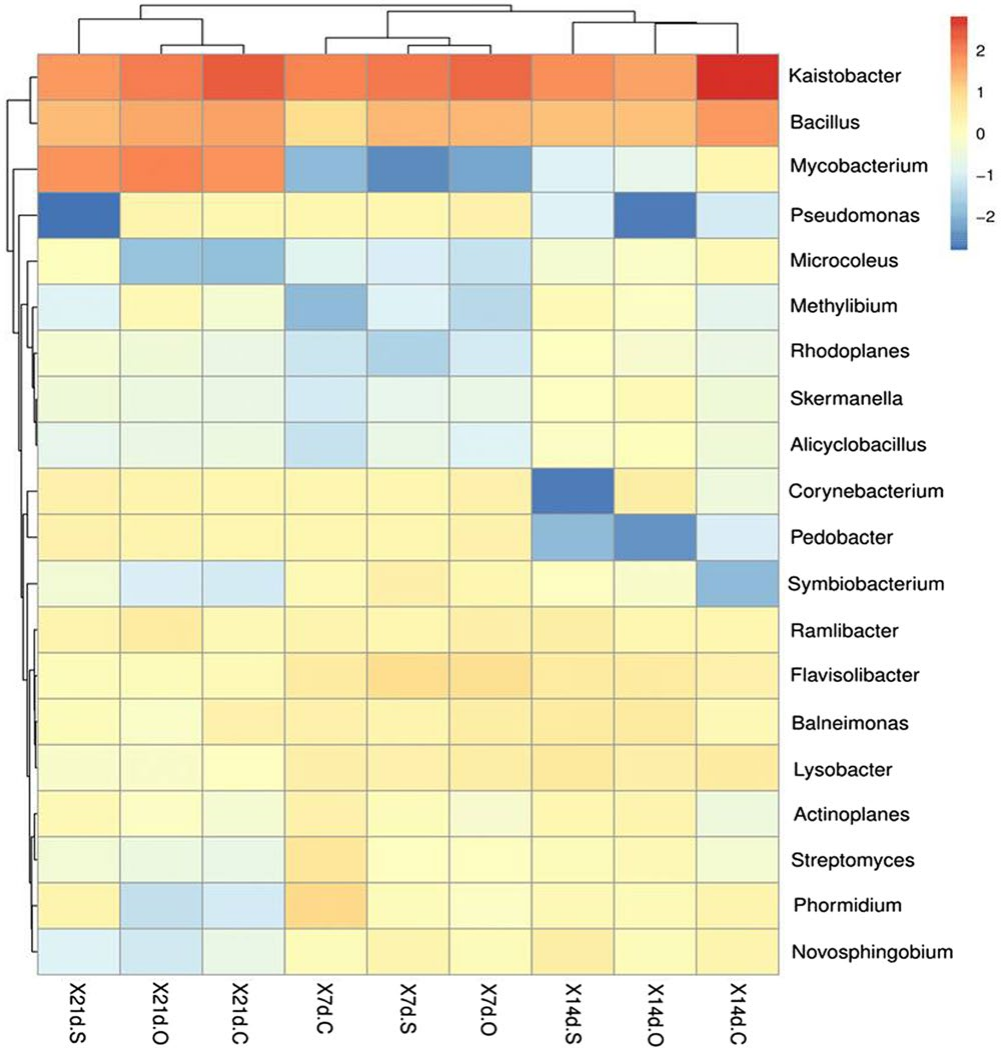
The heat map in relative abundances of top 20 most abundant genera in different cultivation times.

**Figure 6:**
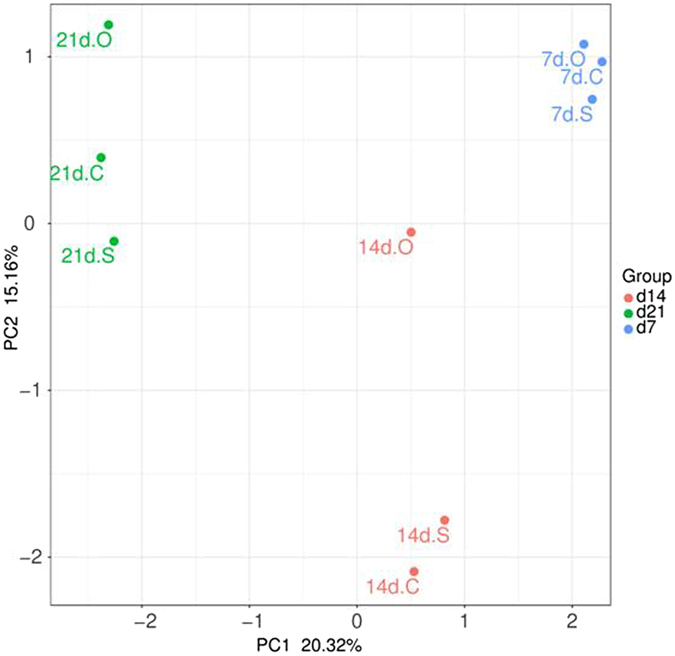
PCoA of microbial community composition.

To visualize the effects of the root exudate components on the bacterial community composition at the genus level, heat map analysis of the top 20 genera was performed. As shown in Fig. 5, nine samples were separated into three groups by the incubation period based on their relative abundances. Principal coordinates analysis (PCoA) was used to evaluate the differences among the bacterial communities. PCoA revealed that three samples were similar at 7 days but separated at 14 and 21 days along principal component1 (PC1, explained 20.3% of the variance) and principal component2 (PC2, explained 15.2% of the variance). This indicated that significant differences were observed in the bacterial communities among the three treatments in the soils incubated for different periods when the root exudate components were added (Fig. 6). Similarly, nonmetric multidimensional scaling analysis (NMDS) indicated a significant difference in the bacterial community composition in this study (Fig. S3). These results indicated that the soil bacterial communities were influenced by the amended substrates and the cultivation time.

## Discussion

Carbohydrates, organic acids, and amino acids are believed to be the main components of root exudates^40^. Due to the diversity and complex composition of root exudates, SAREs were chosen to simulate actual root exudates in this study. SAREs can increase the pyrene degradation in soil. Several possible degradation products of pyrene in control treatment were interpreted according to mass-spectrometry data (Fig. S6). Compared with the control, the organic acids increased the pyrene degradation rate to 14% in 21 days. Similar studies found that LMW organic acids and dehydrogenase activity promoted the removal of PAHs^33^. However, the highest pyrene degradation efficiency, which was higher (reaching 36.1%) than that in the control, was observed when the carbohydrate was added. As a major component of root exudates, the importance of carbohydrates (glucose treatment) has been underestimated.

Enzymes affect microorganisms and may be used as a measure of the activity of microorganisms that are capable of degrading organic pollutants^41, 42^. The urease activity did not significantly change throughout the experimental period, which means that the soil nitrogen utilization did not change (Fig. 2a). This result somewhat reflects that the soil properties have insignificant change following the various chemical added.

Dehydrogenase, which is a typical intracellular enzyme in soil, can catalyze the dehydrogenation of organic matter and is one of the most sensitive parameters for evaluating toxicity. Dehydrogenase is an important enzyme in the decomposition and transformation of cyclic organic compounds and is mainly involved in the formation of catechols as intermediate products of PAH oxidation-dehydrogenation. Thus, increased dehydrogenase activity is beneficial for PAH degradation^43^. In our study, the root exudate components increased the dehydrogenase activity (Fig. 2c). Furthermore, the changes in the dehydrogenase activity were consistent with the removal rates of pyrene (*p* < 0.01) (Fig. S1), indicating that dehydrogenase might be the main enzyme involved in pyrene biodegradation in soil. Therefore, dehydrogenase could be used as an indicator of pyrene biodegradation in this study. The highest dehydrogenase activity was observed in the carbohydrate treatments, which indicated that the carbohydrates in root exudates can significantly increase dehydrogenase activity.

The microbial biomass showed different trends with dehydrogenase during the experiment (Fig. 3a). The biomass decreased at 14 days and then increased at 21 days. According to the results of bacterial growth curve (Fig. S5), it suggested that soil bacteria need some time to acclimate to gain significant growth. Then the biomass decreased and the microbial community structure changed to accommodate the pyrene in soil aged over 12 months. Finally, the microbial biomass increased significantly, which led to the rapid increase in pyrene degradation. In addition, the changes in catalase activity were consistent with the general changes in microbial biomass (*p* < 0.05) (Fig. S2). Because of the detoxification effect, catalase, which widely exists in microorganisms, is often used as an important indicator and plays an important role in the decomposition and transformation of soil organic matter^42^. Therefore, the catalase activity decreased when the microbial biomass decreased, and then the catalase activity increased as the microbial biomass increased. The increased microbial biomass may be explained by the existing microorganisms or by the growth of a new pyrene-degrading bacterium.

Based on the relative PLFA abundances among the different treatments and periods, bacteria were the main microorganisms in the soil (Fig. 3). To describe the changes in the bacterial community more accurately, three groups were selected for further 16S rRNA analysis: the control, carbohydrate and organic acid treatments. At 7–14 days, the toxicity of pyrene reduced the microbial biomass but increased the number of bacterial species (Table 1). The Shannon index showed that the soil microbial diversity was the highest at 14 days. Combined with the changes in microbial biomass, the structure of soil bacteria changed throughout the experiment. Several conventional genera related to pyrene removal were identified in this study (Fig. 4). *Bacillus* was typically associated with major PAH degraders^44^, *Mycobacterium* was a genus of frequently isolated gram-positive bacteria capable of degrading high-molecular-weight PAHs^45–48^, and *Balneimonas* could degrade PAHs but was rarely reported^49^. In the initial stage, most of the pyrene-degrading bacteria were *Bacillus*, and *Mycobacterium* was not responsible for much degradation. However, the populations of *Mycobacterium* increased significantly over 14–21 days and became the major pyrene-degrading bacteria by the end of the experiment. Significant differences in the microbial taxonomic compositions were observed between the beginning and end of the experiment. *Kaistobacter*, *Mycobacterium*, and *Bacillus* were dominant at the end of the experiment. The carbohydrate and organic acid treatments had similar microbial taxonomic compositions (10 dominant bacterial genera) at 14–21 days (Fig. 4). This result may imply that the mechanisms of pyrene biodegradation induced by the addition of carbohydrates were the same as those induced by the addition of organic acids. Moreover, the addition of carbohydrate resulted in the highest abundance of degradation bacteria, followed by organic acid.

We observed the responses of microbial communities to the additions of root exudate components. Changes in the bacterial communities in soil amended with artificial root exudate solutions were revealed by both PLFA and 16S rRNA analyses. The addition of the carbohydrate, followed by the organic acids, resulted in the highest abundance of degradation bacteria. Why the addition of root exudate components results in a significant community shift in contaminated soil remains unknown. However, the PAH degraders increased when the root exudate components were added. These results could indicate the potential importance of root exudate components in influencing the structure of soil bacterial communities during the remediation of contaminated soil, and would advance our understanding of the interaction mechanisms about the role of the components of root exudates on pyrene biodegradation. Meanwhile, knowing which components of root exudates enhance pollutant degradation can aid in the selection of suitable remediation plants.

Root exudates are the most important factor that leads to microbial changes in the rhizosphere and enhances the biodegradation of organic pollution. Root exudates can result in distinct microbial community shifts by altering the microbial catabolic gene expression or the metabolic status and/or by selecting for specific microorganisms, such as degradation bacteria^15^. Some evidence has indicated that LMW organic acids increase the bioavailability of organic pollutants by promoting desorption from the soil matrix^23–25^. However, the concentrations of LMW organic acids in such sorption studies are often much higher than those in normal soil solutions; thus, these mechanisms should be interpreted cautiously^15, 23^. Meanwhile, the results of the desorption experiment showed that the pyrene extracted did not increase at the concentration of 50 mg/kg and 500 mg/kg of simulating compounds (Fig. S4). Although the organic acid treatment resulted in favorable pyrene removal efficiencies, the degradation of pyrene was higher when the carbohydrate was added than when the organic acids were added. As carbon and nutrient sources, it is widely known that carbohydrates significantly increase soil microbial biomass. High nutrient concentrations favor the biodegradation of organic pollutions^50, 51^. The microbial taxonomic compositions in the organic acid treatments were similar to those in the carbohydrate treatment, which could lead to the same induction mechanism. Therefore, carbohydrates and organic acids can be used as carbon and nutrient sources to increase soil microbial biomass, especially for degradation bacteria, to promote the degradation of pyrene. In all treatments, the carbohydrate had the greatest effect; therefore, the effects of carbohydrates cannot be neglected when studying the effects of root exudates. The carbohydrate not only increased microbial dehydrogenase activities but also altered microbial communities and increased the abundance of degradation bacteria. The results of this study provide insight into the functions of root exudate components in the remediation of organic pollutants. The carbohydrates in root exudates should not be ignored in future studies.

## Materials and Methods

### Chemicals

Pyrene was purchased from AccuStandard (NewHaven, USA). All of the organic solvents were HPLC grade, and the silica gel and anhydrous sodium were purified in advance. The other chemicals used in this study had analytical or chromatographic purity and were purchased from Aladdin Chemical Reagent Co., Ltd., China.

### Spiking of soil with pyrene

Soil samples were collected at a depth of 0–20 cm on the farm of Zhejiang University. The soil had a silty loam texture consisting of 18.1% sand, 68.1% silt, and 13.8% clay, as determined using a laser particle analyzer (Malvern MAM-5005, England). In addition, the soil contained 0.69% organic carbon and had a pH of 6.6. All of the soil samples were air dried and then passed through a stainless steel 10-mesh sieve. A soil sample (approximately 50 g) was spiked with pyrene dissolved in acetone and then mixed completely after the acetone had volatilized. Next, the spiked soil was mixed well with approximately 450 g of clean soil and then mixed completely with approximately 4500 g of clean soil. All of the spiked soil samples were aged for more than 12 months. The concentration of pyrene in the spiked soil was 120.2 ± 1.76 mg/kg.

### Degradation experiment

SAREs were obtained using a simplified method based on a previous study^52^. Root exudates were prepared by mixing 150 mM glucose, 25 mM oxalic acid, 25 mM malic acid, and 37.5 mM serine, which were filter-sterilized by passing through 0.22 μm filters. Spiked soil was placed into culture dishes, and five treatments were applied: (1) distilled water, (2) SAREs, (3) a carbohydrate (glucose), (4) organic acids (succinate and malic acid), and (5) an amino acid (serine). In treatments 2–5, the added TOC concentration was 50 mg/kg soil. The contents of each dish were added to 50 g of the contaminated soil (dry weight), and the soil was then placed into an incubator. Killed and dark controls were performed containing 0.05% NaN_3_^58^. The soil moisture content was maintained at approximately 60% of the field water-holding capacity (WHC). Each treatment was replicated nine times. Every seven days, triplicate samples from each treatment were collected and analyzed. Soil samples from each culture dish were homogenized and divided in two for chemical and biological analyses.

### Pyrene extraction and analysis methods

Soil samples were freeze dried and then passed through 100-mesh sieves prior to analysis. Pyrene was extracted from the soil samples using an ultrasonic-assisted solvent extraction technique, and the pyrene concentrations were determined using high-performance liquid chromatography (HPLC) per the methods described by Gao *et al*.^53^.

### Soil enzyme analysis

The activities of three soil enzymes, i.e., urease, catalase and dehydrogenase, were determined. The activity of urease was measured using the method described in a previous study^54^. Briefly, a 10% aqueous solution of urea was added to soil as substrate for urease. The soil was incubated for 24 h at 37 °C; after filtration, sodium phenolate and sodium hypochlorite solutions were added to the sample, and the absorption of the filtrate was measured at 578 nm. The catalase activity was determined using a reported method^55^. The soil was placed in a 0.3% aqueous solution of H_2_O_2_ and then shaken at 30 rpm for 10 min at 20 °C. Then, the indicator reagent, which included a specific concentration of peroxidase, was added to the soil filtrate, which was left to stand for 5 min before measuring its absorbance at 505 nm. The dehydrogenase activity was assayed by measuring the absorbance at 485 nm following the addition of triphenyltetrazolium chloride (TTC) as the substrate, which was converted to triphenylformazan (TPF)^54^.

### PLFA analysis

The method used to extract and purify soil microbial PLFAs was described previously^56^. Briefly, soil samples were extracted using a chloroform/methanol/phosphate buffer. Transesterification was conducted using a KOH methanolic solution after purification and on with a SPE column. Finally, the solution containing fatty acid methyl esters (FAMEs) was stored until analysis on a gas chromatograph (GC) equipped with a MIDI Sherlock microbial identification system. PLFAs were identified and categorized into three groups: bacteria, fungi, and actinomycetes^39, 56, 57^. The total concentration of PLFAs was used as the relative soil microbial abundance.

### Soil DNA extraction, 16S rRNA gene amplification and high-throughput sequencing

Representative samples were selected for further sequencing. Soil DNA was extracted from 250 mg samples that had been homogenized as described previously using a PowerSoil DNA Isolation Kit (MoBio Laboratories Inc., Carlsbad, USA). Later, PCR amplification and high-throughput sequencing were conducted by GUHE INFO (Hangzhou, China). Briefly, the V3–V4 region of the 16S RNA gene was amplified using the following universal primers: forward 5′-ACTCCTACGGGAGGCAGCAG-3′ and reverse 5′-GGACTACHVGGGTWTCTAAT-3′. Amplification was performed using an XP Cycler (Bioer, Hangzhou, China) with the following conditions: pre-denaturation at 98 °C for 30 s; 30 cycles of denaturation at 98 °C for 15 s, annealing at 58 °C for 15 s and extension at 72 °C for 15 s; final extension at 72 °C for 1 min; and holding at 4 °C. The products were purified by agarose-gel-electrophoresis and recovered using an AXYGEN Gel Extraction Kit. The products were quantified and sequenced using Qubit 2.0 (Q32866, Life Tech) and the Illumina MiSeq platform.

### Desorption of pyrene as affected by root exudate components

Approximately 2 g of the spiked soils was weighed and placed into 42-mL glass centrifuge tubes. 40 mL of solution of each of the root exudate components (equivalent to 50 and 500 mg TOC/kg) was added containing 0.05% NaN_3_ to inhibit microbial activities based on a previous study^58^. The tubes were shaken in the dark at 200 rpm on a gyratory shaker to achieve an apparent equilibrium desorption. Then, the vials were centr only a small percentage of the organic pollutants ifuged at 3000 rpm for 10 min. The supernatant was taken out and the soil left in the tube was freeze-dried. Pyrene in the soil and supernatants were extracted and analyzed using a reported method^59^. All equilibrium concentrations of pyrene in the solutions were below their aqueous solubility.

### Statistical analysis

Statistical analyses, including analysis of variance (ANOVA) and significance testing, were conducted using SPSS 18.0. Origin Pro 8.0 software was used to produce the figures.

## Electronic supplementary material

Supplementary information
