# Supplementary material for: The role of artificial root exudate components in facilitating the degradation of pyrene in soil
Source: Sci Rep. 2017 Aug 2;7:7130. doi: 10.1038/s41598-017-07413-3 (PMC5541004; doi:10.1038/s41598-017-07413-3)
Supplement: Supplementary file 1 — Supplementary information [file 41598_2017_7413_MOESM1_ESM.pdf]

Supplementary Information

The role of artificial root exudate components in facilitating the degradation of pyrene in soil

Hainan Lu [a,b], Jianteng Sun [a,b], Lizhong Zhu [a,b,*]

[a] Department of Environmental Science, Zhejiang University, Hangzhou, Zhejiang, 310058, China

[b] Zhejiang Provincial Key Laboratory of Organic Pollution Process and Control, Hangzhou, Zhejiang, 310058, China

* Corresponding author: Lizhong Zhu

Phone/Fax: 86 57188273733.

E-mail: zlz@zju.edu.cn

Contents

Table S1. Relative abundance (%) of dominant bacterial lineages in soils revealed by 16S rRNA clone library analysis at the phylum level.

Figure S1. Correlation between dehydrogenase activities and degradation rates of pyrene.

Figure S2. Correlation between catalase activities and total PLFAs.

Figure S3. NMDS of microbial community composition.

Figure S4. Desorption of pyrene from soils as the concentrations (50 and 500mg/kg) of the root exudate components.

Figure S5. Bacterial growth curve in different cultivation times.

Figure S6. Intermediate metabolic products of pyrene degradation from blank sample analyzed by gas chromatography (GC) mass spectrometry (MS).

Materials and methods

Bacterial growth curve

The bacterial growth curve was carried out by measuring the content of soil DNA. Soil samples were collected at 3, 6, 9, 12, 15, 18, and 21 days. Soil DNA was extracted from 250 mg samples that had been homogenized as described previously using a PowerSoil DNA Isolation Kit (MoBio Laboratories Inc., Carlsbad, USA). Total DNA was measured using NANODROP 2000 (Thermo scientific, USA).

Identification of the intermediates

Briefly, control soil (approximately 10 g) was extracted by 90 ml phosphate buffer. Pyrene dissolved in acetone was added to the flasks. After acetone evaporated, the soil solution was added into the flasks. The flasks were incubated on a rotary shaker (200 rpm) at 30 ℃ in dark for 2 days. The pH of the soil solution was adjusted to 2 with HCl. and the culture was extracted three times with equal volume of ethyl acetate. All the collected ethyl acetate was evaporated under a gentle nitrogen stream in fume hood. 80 μL anhydrous pyridine was added to dissolve the residue mixture, then derivatization was performed using 20μL N-Methyl-N-(trimethylsilyl) trifluoroacetamide (MSTFA) at 60 ℃ for 30 min. The culture extract was subjected to GC-MS (Agilent 5977B-7890B, USA) analysis. HP-5MS capillary column (ID 0.25 mm, length 30 m and 0.25 μm film thickness) was used with helium as the carrier gas. The oven temperature was initially kept at 50 ℃ and was increased to 310 ℃ at a rate of 5 ℃ /min. The injector and detector temperatures were set at 250 ℃ and 280 ℃, respectively. The mass spectrometer was operated at 70 eV of electron ionization energy, mass range 50-600 m/z.

Table S1 Relative abundance (%) of dominant bacterial lineages in soils revealed by 16S rRNA clone library analysis at the phylum level

	**7d.C**	**7d.O**	**7d.S**	**14d.C**	**14d.O**	**14d.S**	**21d.C**	**21d.O**	**21d.S**
Proteobacteria	25.4±1.4	31.5±2.1	25.8±2.6	23.7±3.0	28.5±1.3	27.4±1.8	28.4±4.0	29.1±3.5	24.1±0.7
Acidobacteria	24.1±2.0	22.9±0.2	22.3±1.3	22.0±3.8	17.7±3.6	20.6±1.9	16.5±5.3	16.4±1.2	17.3±0.1
Actinobacteria	14.1±3.1	12.0±1.3	14.2±0.9	15.7±0.2	16.2±3.1	14.1±3.0	18.1±2.5	21.2±0.1	20.6±2.5
Chloroflexi	10.4±1.2	9.3±1.4	10.4±0.2	11.3±0.9	10.3±0.8	10.9±0.1	9.0±2.3	7.5±0.9	10.1±0.6
Gemmatimonadetes	10.0±0.3	9.9±0.7	10.5±0.8	13.2±0.0	11.9±0.5	11.9±1.2	11.8±2.6	9.8±0.2	11.2±1.5
Firmicutes	3.7±0.2	5.1±0.8	4.7±0.7	4.3±0.5	4.7±0.4	5.1±2.0	3.9±1.8	5.1±0.3	5.5±1.8
Cyanobacteria	4.9±1.0	2.2±0.1	4.3±2.2	3.3±0.8	2.9±0.5	2.2±0.1	4.4±2.3	4.0±1.2	4.1±0.4
Bacteroidetes	2.7±0.3	2.9±0.5	3.1±0.5	1.8±0.6	2.7±0.5	1.7±0.1	1.9±0.0	2.0±0.1	1.6±0.3
TM7	1.0±0.1	1.0±0.1	1.6±0.26	1.5±0.6	1.2±0.5	2.3±0.4	1.3±0.1	1.2±0.1	1.8±0.0
OD1	1.0±0.3	0.8±0.1	0.8±0.1	0.8±0.2	1.1±0.1	1.0±0.2	1.6±0.5	1.0±0.0	1.2±0.1
Others	2.7±0.4	2.3±0.1	2.3±0.4	2.5±0.2	2.8±0.4	2.6±0.1	2.5±0.4	2.6±0.1	2.4±0.4

7d: 7 days, 14d: 14 days, 21d: 21 days, C: control, O: organic acid, S: carbohydrate, “Other bacteria” stands for unclassified bacterial genera presented in the soil.

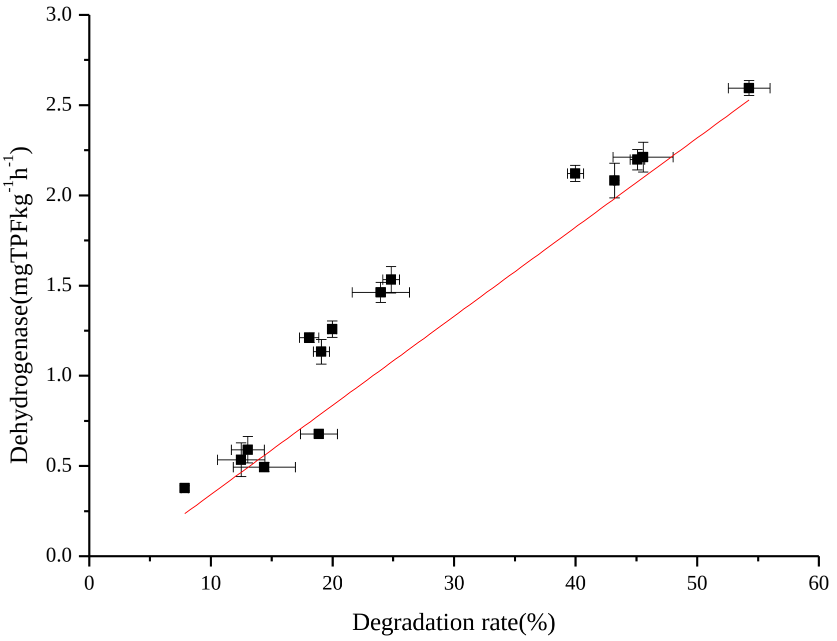

Figure S1. Correlation between dehydrogenase activities and degradation rates of pyrene. The equation is: y=-0.153 + 0.0494x (r^2 = 0.83, p< 0.01), where y is soil dehydrogenase activities and x is degradation rate of pyrene. Error bars represent standard deviations (n=3).

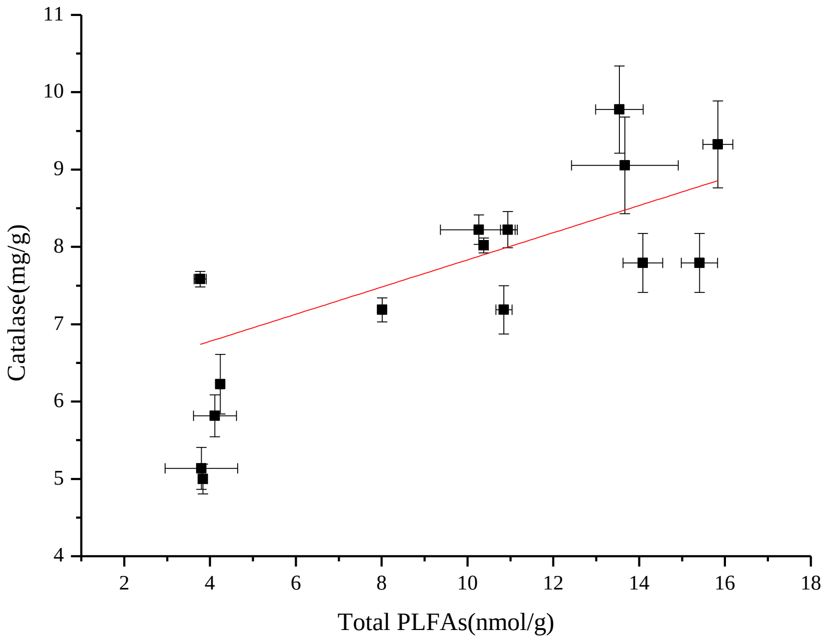

Figure S2. Correlation between catalase activities and total PLFAs. The equation is: $y=6.07 + 0.176x$ ($r^2 = 0.334$, $p< 0.05$), where y is soil catalase activities and x is the total PLFAs of soil. Error bars represent standard deviations (n=3).

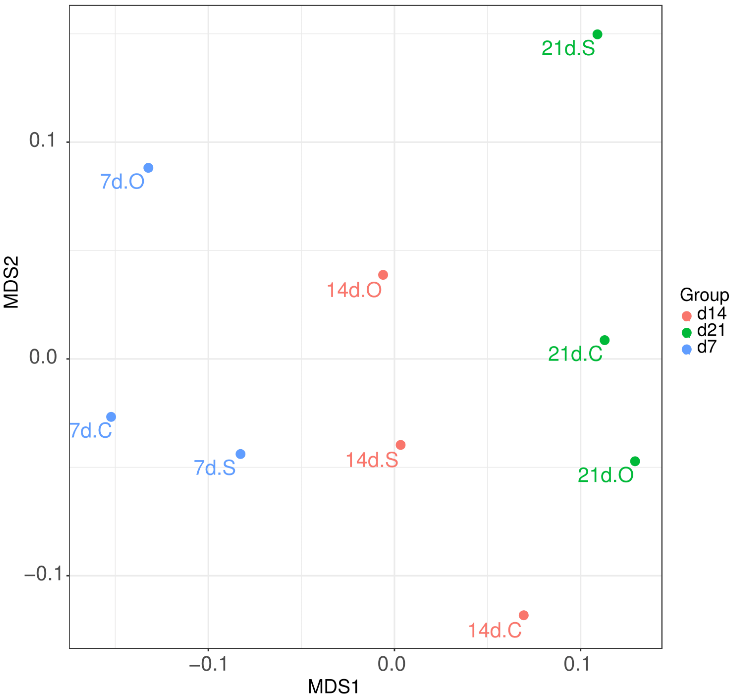

Figure S3. NMDS of microbial community composition. 7d: 7 days, 14d: 14 days, 21d: 21 days, C: control, O: organic acid, S: carbohydrate.

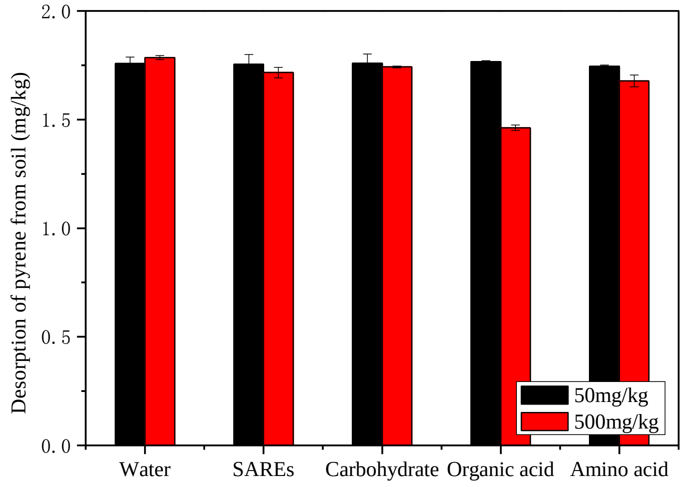

Figure S4. Desorption of pyrene from soils as the concentrations (50 and 500mg/kg) of the root exudate components. Error bars represent standard deviations (n=3).

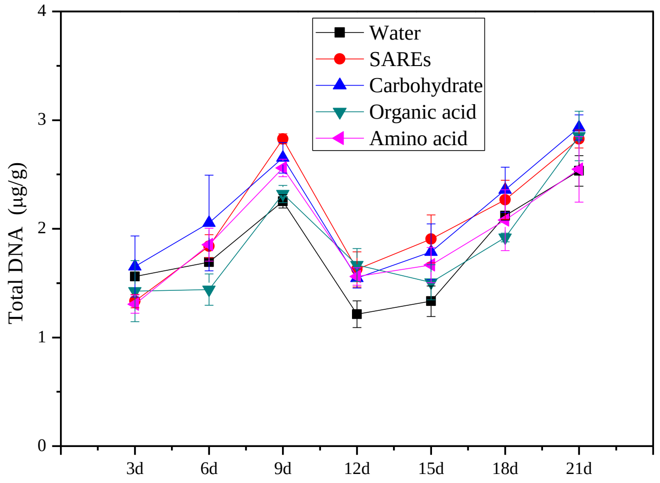

Figure S5. Bacterial growth curve in different cultivation times. Error bars represent standard deviations (n=3).

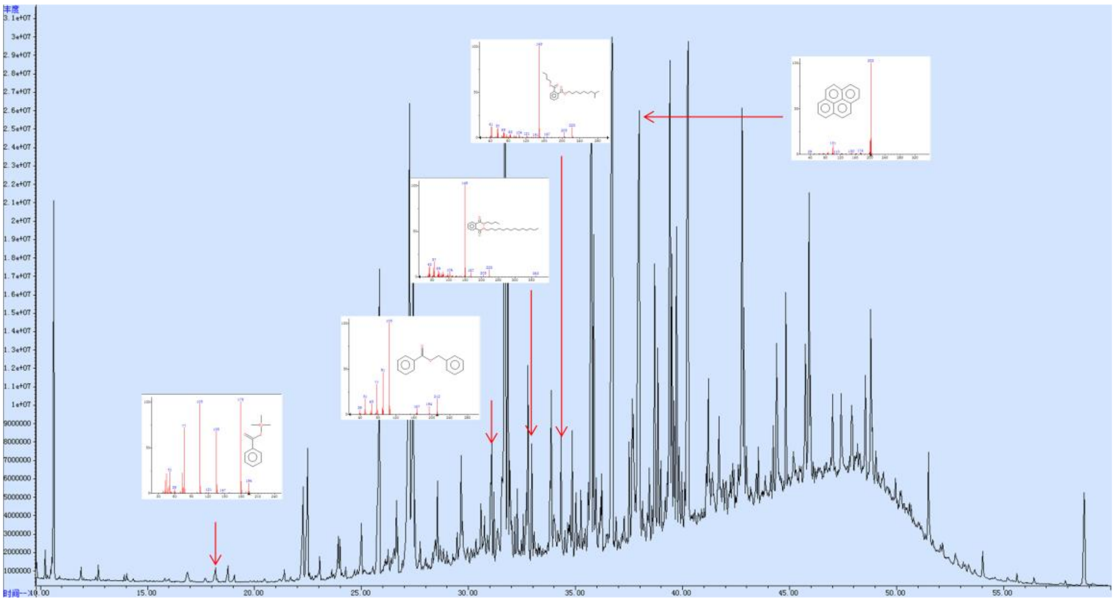

Figure S6. Intermediate metabolic products of pyrene degradation from blank sample analyzed by gas chromatography (GC) mass spectrometry (MS).
